# A Blockchain-Driven Supply Chain Finance Application for Auto Retail Industry

**DOI:** 10.3390/e22010095

**Published:** 2020-01-13

**Authors:** Jingjing Chen, Tiefeng Cai, Wenxiu He, Lei Chen, Gang Zhao, Weiwen Zou, Lingling Guo

**Affiliations:** 1 Department of Computer Science, Zhijiang College of Zhejiang University of Technology, Shaoxing 312000, China; joyjchan@gmail.com (J.C.); ctf@zjc.zjut.edu.cn (T.C.); wenxiuhe@zjut.edu.cn (W.H.); 2 Sunyard System Engineering Co., Ltd., Hangzhou 310000, China; gang.z@sunyard.com; 3 Huawei Noah’s Ark Lab, Hong Kong 999077, China; lc.leichen@huawei.com; 4 Department of Computer Science, Hong Kong Baptist University, Hong Kong 999077, China; zwilman@gmail.com; 5 Department of Chemical Engineering, Zhejiang University of Technology, Hangzhou 310000, China

**Keywords:** blockchain, supply chain finance, auto retail industry, SMEs

## Abstract

In this paper, a Blockchain-driven platform for supply chain finance, BCautoSCF (Zhi-lian-che-rong in Chinese), is introduced. It is successfully established as a reliable and efficient financing platform for the auto retail industry. Due to the Blockchain built-in trust mechanism, participants in the supply chain (SC) networks work extensively and transparently to run a reliable, convenient, and traceable business. Likewise, the traditional supply chain finance (SCF), partial automation of SCF workflows with fewer human errors and disruptions was achieved through smart contract in BCautoSCF. Such open and secure features suggest the feasibility of BCautoSCF in SCF. As the first Blockchain-driven SCF application for the auto retail industry in China, our contribution lies in studying these pain points existing in traditional SCF and proposing a novel Blockchain-driven design to reshape the business logic of SCF to develop an efficient and reliable financing platform for small and medium enterprises (SMEs) in the auto retail industry to decrease the cost of financing and speed up the cash flows. Currently, there are over 600 active enterprise users that adopt BCautoSCF to run their financing business. Up to October 2019, the BCautoSCF provides services to 449 online/offline auto retailors, three B2B asset exchange platforms, nine fund providers, and 78 logistic services across 21 provinces in China. There are 3296 financing transactions successfully completed in BCautoSCF, and the amount of financing is ¥566,784,802.18. In the future, we will work towards supporting a full automation of SCF workflow by smart contracts, so that the efficiency of transaction will be further improved.

## 1. Introduction

Under the influence of the continuous international financial crisis, the development of the world economy is facing unprecedented difficulties [1,2]. It is essential and urgent to establish a fully decentralized, reliable, and sustainable financial platform without influence from any institutions or individuals. Due to the lack of trust among related parties, the traditional financial mode no longer adapts to the rapid development of society. With a wave of the Internet technology, the financial innovations are constantly emerging. Bitcoin—the first crypto currency, launched in 2009. Its underlying technology is Blockchain [3]. Babich and Hilary emphasize its full potential with respect to operations management [2]. As one of the promising application fields of Blockchain technology, SCF strengthens cooperation and reinforces connections among various enterprises, and accelerates cash flows within the SC [4,5]. Blockchain technology (BCT) reasserts the mechanism of the value exchange and the way of information exchange via the Internet, leading to a new collaboration among participants in SC networks [5,6]. It is a huge waste of manpower and physical space to verify and preserve these traditional paper records of trading [7,8]. In contrast, digital document management system and entry retrieval system will help reduce the workload and the difficulty of tasks, and further help realize the procedural management and cost control [6,9].

For the Auto retail industry, participants in the SC (e.g., manufacturers, suppliers, carriers, warehouse, terminal buyers, and funding providers, etc.) had limited peer-to-peer communication with regards to their own concerns in traditional setting [10]. One of the biggest hurdles in information exchange is the “trust”. In addition, there are high risks of tampering while involving in debit notes, contracts, and warehouse receipts. Actually, in a centralized information environment, it is easy to tamper a record [8]. Moreover, it is costly to perform efficient custody, especially difficult to verify the authenticity of an invoice/a receivable [3,11]. Contrarily, BCT promises honesty and allows secure authentication of logistics and information circulation in a SC network [12,13]. With the development of BCT, its applications in SC fields are rapidly emerging [13]. This paper implements a Blockchain-driven SCF platform, which aims to ensuring the trust among shareholders and reducing the financing cost for the Auto retailer industry.

This study contributes to the existing literatures in following aspects: (1) We extend the work of Pournader [14], further identify existing barriers that negatively influence SCF, and explore theoretical solution enabled by BCT built-in mechanisms to overcome these barriers. (2) Aiming to verify our solution, we implement a BCT-driven SCF platform and put it into practical use. In this paper, we comprehensively present its design, models, and workflows, which are first-hand practical materials for academic and industry when applying BCT to SCF.

As an advancement of practice, our contribution lies in establishing an efficient, trustable, and reliable financing platform for SMEs in the Auto retail industry. The analysis towards the operational data indicates that this platform significantly improves the transactions within SC, decreases the cost of financing and speeds up the cash flows for SMEs. Currently, the platform serves over 600 enterprise users (i.e., 449 online/offline Auto retailors, 3 B2B asset exchange platforms, 9 fund providers and 78 logistic services across 21 provinces in China). There are 3296 financing transactions successfully completed in BCautoSCF, and the amount of financing is ¥566,784,802.18. 

The remainder of this paper is organized as follows: Section 2 describes the related work. Section 3 proposes the conceptualized design of Blockchain-driven SCF. Section 4 presents the system implementation. The findings and discussion are presented in Section 5. Conclusion and future direction are introduced in Section 6.

## 2. Materials

### 2.1. Blockchain Technology

Recent Blockchain research activities, which are first proposed by “Satoshi Nakamoto” [7], have been deeply committed to solving practical problems in the industry and integrating with the regulatory authorities. This section will introduce the principal features of the BCT.

#### 2.1.1. Peer to Peer Network

A peer to peer network under distributed network architecture was first reported for sharing hardware resources among network members (i.e., processing power or storage capacity) by Schollmeier et al. [8]. These resources can be shared among other peers without the participation of central entity, meanwhile, providing services and content, such as file sharing, storage, and sharing of collaborative workspaces. Likewise, the current centralized system; transactions in the blockchain system are broadcast and stored throughout various participants in the network. Owning to the transparent operations without user intervention, such transactions will be completed in a very short time.

#### 2.1.2. Consensus Mechanism

The first priority is the “trust issue” in the Blockchain-driven system that is, maintaining a single record history between different participants in the network that store transactions [7]. To enable the peers to reach a consensus for the instant status of the shared ledger, and the undisputed group consensus mechanism is the foundation of BCT. A consensus mechanism refers to a way to reach an agreement. All peers in a Blockchain network have to act under certain conditions and reach a collective agreement. However, each Blockchain context requires specific consensus algorithm.

“Bitcoin”, a global digital currency known in the past for its Blockchain system, employs the proof of work (PoW) consensus mechanism [15]. A difficult computational problem (i.e., a hash computation problem) needs to be addressed when creating a new transaction block. Meanwhile, consensus mechanism is based on such a hard to solve but easy to verify problem, which avoids the necessity for other peers to redo the PoW for transaction acceptation. With this mechanism, each block is connected with the previous block to form a long block chain. Proof of stake (PoS) is designed to deal with the main shortcoming of the PoW. The PoS is a new type of concept where every individual can mine or even validate new blocks only based on their coin possession. Delegated proof of stake (DPoS) is a refined version of the typical PoS [15]. It is fast and efficient. Proof of elapsed time (PoET) is designed for permissioned Blockchain network where you will have to get permission for accessing the network. These permissions networks need to decide on the mining rights or voting principles. It ensures a secure login into the system, as the network requires identification before joining the miners [16]. Practical Byzantine fault tolerance (PBFT) is always designed for practical use and extremely easy to implement, and it replicates the system but gets rid of the main Byzantine general problem. PBFT possesses a proved advantage over all other consensus mechanisms [17]. In this study, we use PBFT to implement the consensus mechanism.

#### 2.1.3. Permissions

Generally, Blockchain can be grouped into public Blockchain and permissioned Blockchain [9]. The public Blockchain uses security measures to limit the condition and range of using strict responsibility to avoid abuse happening. For these reasons, some financial institutions are aware of the need to develop unique systems with both private networks and verification nodes, which is known as permissioned ledgers. Such permissioned ledger exhibits great advantage and application foreground due to the omitting of work verification, and a business scenario, which involves heavy transaction throughput. Essentially, it is not a completely open and decentralized network secured by “puzzle” mechanism [9], but a system where the access permission is severely limited, and the modified right of Blockchain is restricted to specific users. Permissioned Blockchain can be further classified into consortium Blockchains and fully private ones. Consortium Blockchain is one distributed ledger system with the consensus process controlled by a selected group of peers. The Blockchain query permission can be public or be granted to selected participants. The permissions of fully private Blockchains remain centralized to a specific organization, and query permissions can be public or be granted to selected participants. Since operations stuff in the system includes data management and other internal uses for a single organization. In this study, with regards to the application context of SCF, we use consortium Blockchain to build our underlying infrastructure.

#### 2.1.4. Smart Contract

The concept of “Smart contract” is first described and named by Szabo in 1990s, which digital assets are operated by a program executing arbitrary rules [18]. To be specific, in BCT, it is an event-driven program clip, which runs on a shared ledger and takes custody over digital assets on the Blockchain. Smart contract is first implemented in the Ethereum project, which is a popular BCT project with a built-in turning-complete programming language [15,19]. It allows users to create programs, commands, and decentralize applications to execute arbitrary rules such as state transition functions, ownership, etc. [19]. Previous works [19,20,21] proved that financial contracts and legal rules were eligible of being described and presented in computerized form.

In BCT, a smart contract is designed to react with those events (called “inputs”) and ensure logics are fully accurately executed (called “outputs”) across untrusted entities without any modification (called “abuse”) by any party in Blockchain networks [6]. Its principle features that distinguish itself from traditional ones are as follows: (1) It is decentralized: A smart contract is registered to a Blockchain and is thus distributed and self-executed across the peers in the Blockchain network; (2) It is automated: Once a smart contract was activated and launched, it would run by itself and there is no need to require further intervention; (3) It is self-sufficient: A smart contract is capable of independently marshaling resources in the Blockchain network. For instance, in a SC application, a smart contract is capable of issuing digital equity and further spending them as required, or raising funds by providing services [6,9,22]. 

In this case, the objectives of a smart contract are to meet common contractual conditions (e.g., due date, payment terms), avoid exceptions and remove the need for trusted intermediaries. First, the related trading parties negotiate the predefined conditions, the physical assets under custody and common obligations for each party in SCF. Then, the smart contract is registered on a Blockchain. If these predefined conditions are met, it will trigger the execution of the smart contract, and the program clip then automatically dictates the transfer of physical/ digital value accordingly. 

### 2.2. Supply Chain Finance

SCF can be defined as the refining of financing modes and the integration of financing processes among suppliers, customers, and other roles to increase the value for all participants within SC [2]. SCF aims to optimize the financial structure and cash flow within the SC. Researchers also emphasize its objective of optimizing financing across borders to speed up the cash flows and lower the cost of capital [2,5,11]. Technically, SCF is a series of new finance products designed for requiring a cheaper form of financing, which is mainly provided by financial institutions. To generate liquidity and improve working capital, these institutions use documents, orders, and contracts for inter-firm transactions and further help them obtain better payment terms. There are three key components consisting SCF: (1) Working capital management, (2) open account trade, and (3) technology offerings [3]. SCF is becoming more and more critical for SMEs [11,12,23]. The traditional form of financing, for instance, trade credit (TC) from suppliers is dominant. However, the extension of TC is directly subjected to the bargaining power whereby weaker suppliers will be forced to expand the payment period or delay the repayment, which always raises risk and disruption within the SC [11,23]. Therefore, there is an urgent need of the better management and optimization of working capital within the SC. From this perspective, SCF is capable of creating multi-win situations for participants in SC.

SCF integrates financing and risk mitigation techniques to enhance the supply chain process, reduce the operational cost, and monitor the floating risk. SCF is usually applied to open account transaction. The visibility and traceability of these underlying information flows among these finance parties is necessary for such financing arrangements [23,24]. The major forms of SCF include: (1) Factoring: An emerging comprehensive financial service, that consists of commercial credit information, trade finance, receivables, and credit risk deposits. It is a form of receivables purchase, where goods and services are sold at a discount price to a finance provider. (2) Order financing: A classical self-liquidating financing technique. (3) Inventory financing: A specific loan for fund inventory purchases, and the purchased inventory acts as collateral to secure the loan. Meanwhile, term loans or credit lines can also be used to purchase inventory. Nowadays, invoice-based financing takes the largest share (up to 90% market share), meanwhile, inventory financing and pre-shipment financing just take very limited market shares and are restricted to certain industries [21]. 

As emphasized in literatures [21,23,25], technology plays a critical role in SCF: The advancement of application systems and platforms enables businesses to run smoothly and speed up workflows throughout the SC, supporting various kinds of financing scenarios (e.g., reverse factoring, reverse securitization, etc.). However, most of these systems and platforms are centralized and require trusted intermediaries (i.e., trust dependency). The centralized SCF platforms usually face the challenge such as information abuse, record tamper, transaction unverifiable, and etc. Thus, these barriers decrease the willingness to join the platform and increase the transaction costs, and have a negative impact on spreads and the value created for the SC community and its shareholders [26,27]. BCT as a promising decentralized technology, without requiring peer to peer trust, could drive decentralized services that aims to speed up business processes, and make financing less costly, but more efficient [27]. 

### 2.3. Blockchain-Driven Supply Chain Finance Application

The dominant factor of successful SCF application is to enable enterprises to run together and accelerate cash flow throughout the whole supply chain ecosystem [12,21,25]. BCT has changed the way that individuals and organizations exchange information and transfer value via the Internet, and has opened up new application prospects of collaboration between supply chain participants [23]. For instance, a Foxconn cooperative has established a Blockchain-based platform, aiming to eliminate barriers and make information sharing accurate, reliable, and secure [26,27,28]. The traditional letters of credit always require manual comparison of different paper trade finance documents for compliance checks, leading to high labor costs and time costs. As a new technology applied in SCF, BCT is widely used in the private and public sectors in the form of concept verification prototype. Recently, the US government has built up an open source tool to develop and test BCT services for public purposes [17,23]. Yang introduced the Blockchain-based maritime shipping digitalization and also discussed the future improvement directions in the Blockchain technology [21].

A SCF application is attractive and effective for its implementation based on two main baselines: (1) Information exchange is secure, verified, and trustable throughout the Blockchain in real time, so that all members of the supply network can access this information any instant. (2) Automatic validation and transaction execution can be implemented when certain criteria are triggered by a smart contract [23]. Driven by the BCT, the workflows in traditional SCF can be significantly refined: Smart contracts can react to SCF events and could be applied to physical assets (i.e., collaterals) represented outside a Blockchain (i.e., SC networks). 

## 3. Proposed Solution 

The adoption of SCF solutions relies on the capacity of the solution to establish reliable custody mechanism on multilateral trades and the willingness (i.e., trust) of the relevant parties to join the complex trades, where consortium is assembled. Fast and reliable financing from the BCT-driven trust mechanism could create more stable SCF ecosystems. By introducing BCT and leveraging other technologies (e.g., cloud computing, the Internet of Things), we established an integrated SCF platform for the Auto retail industry. The platform provides a wide array of functions including logistics/warehouse management, funding/credit service management, purchase order management, and platform administration. It works closely with finance institutions to provide inventory financing and purchase order financing. BCautoSCF not only possess market penetration and logistics capabilities, but also help customers find new growth points and cut down the overall trade cost in SC operations by coordinating the information and business flows of customers’ upstream and downstream operations. To some extent, BCautoSCF takes on the role of both the coordinator and orchestrator of the SC operations.

### Participants

**Figure 1:**
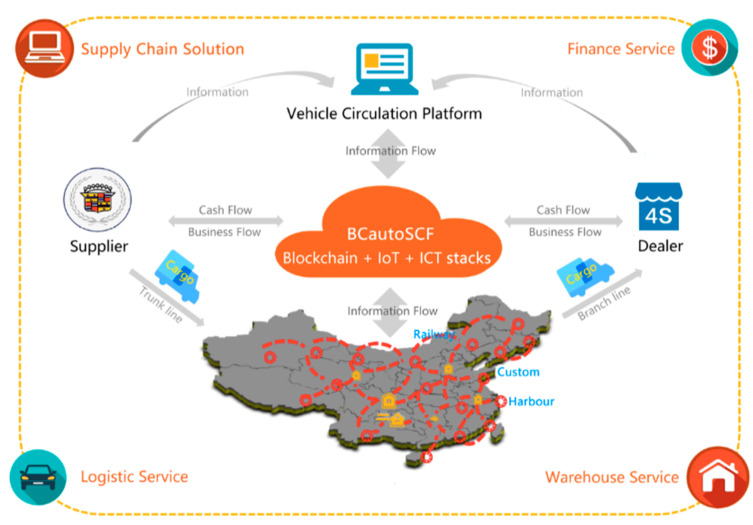
The coordination and orchestration among roles in the Blockchain-driven platform for supply chain finance (BCautoSCF) platform.

There are various participants in BCautoSCF concerning different activities in SCF. The finance institutions (e.g., banks, etc.) are the funding source in this business. In the manufacturer industry, the close collaboration with finance institutions enables better support into its operation and development; and, at the same time, the SCF service also represents a new income source for the finance institutions. The logistics service provides reliable transportation and warehouse service, which could significantly reduce the vehicle delivering cost and ensure the vehicle safety on the road. For suppliers, they can significantly ease their cash flow pressure. For retailers, they can obtain funding to extend their market channels and ease their pressure of full payments for auto manufacturers. In addition, inside the supply chain ecology, efficient logistics service further lowers the overall transaction cost, resulting in a better pricing of vehicles they sell. BCautoSCF also provides financing service to the secondary distributors and other types of small retailers, and helps them secure goods supply and enjoy the benefit of volume discounts. BCautoSCF acts as the fourth-party custody platform. It is capable of full custody on the cash flow, information flow, logistic flow and business flow in every transaction, and further combines them to form time-stamped transaction records stored in the shared ledger. It brings fully transparent transaction details to all granted participants. Each participant in BCautoSCF could significantly benefit (e.g., reduce cost, accelerate cash conversion, create new income source, etc.) from the completely equal collaboration with each other and build-in trust mechanism (illustrated as Figure 1).

## 4. Implementation

### 4.1. Functionalities and Workflow

**Figure 2:**
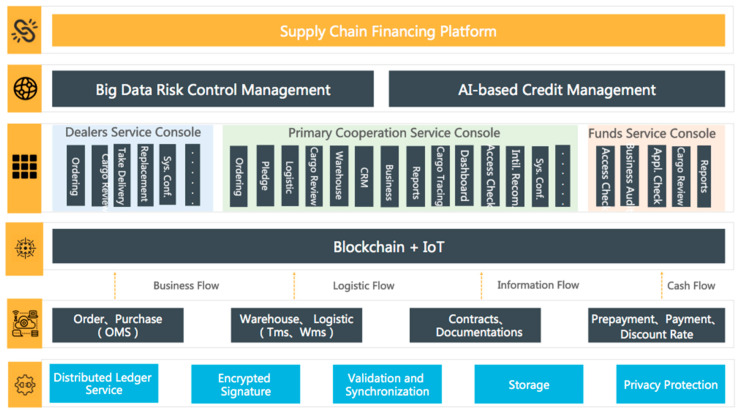
The functionality hierarchy of the BCautoSCF platform.

BCautoSCF is driven by IoT and BCT, where IoT is designated to serve as implementing data acquisition and data circulation while BCT guaranteed that shared and published information is reliable and authentic in a transaction [22,26,27,28]. It is implemented based on a commercial BCT infrastructure (i.e., Xuper, https://xuper.baidu.com, PBFT as consensus algorithm), developed by a vast array of programming technique stacks (e.g., Java, Python, C++, etc.), and runs on the basis of Software as a Service (SaaS). As described in Figure 2, the functionality hierarchy consists of: (1) Underlying protocols. It is used as the fundamentals of trust mechanism, including distributed ledger, encrypted signatures, validation/synchronization, and block-linked storage and privacy security. (2) Custody data. It integrates various data sources to form a time-stamped data stream, including ordering/purchasing, warehousing/logistics, contracts/documents and pre-payment/invoices. (3) Infrastructures. It is the core physical and virtual building to construct the platform, including Blockchain implementation, IoTs, BDS, global positioning system (GPS), radio frequency identification (RFID), image recognition (IR), etc. (4) Business management. It implements a wide type of business management and visualization modules, for instance, vehicle monitoring system, which usually equips IoT garments to ensure that the collateral is under the full custody. The transportation management system and warehouse management system provide reliable logistic service. (5) Third party integration. It connects third party services (e.g., ant credit) for the risk control. (6) User interfaces. It implements the user interfaces based on web technology.

### 4.2. Transaction Tractability

The tractability of collaterals (i.e., vehicles) in providing SCF service to customers is a key advantage distinguishing itself from other platforms. To strengthen the tracking of vehicle dynamics throughout the SCF network, BCT is facilitated in pairs with IoT-transponders (or tags) on the vehicle, which carries different dimensional information acquisition and synchronization through touchless operations. These garments are linked to the backend service, giving a unique and immutable identity. In this way, the vehicles can be easily identifiable, and a specific token could digitally represent a specific vehicle. By using BDS, BCauotSCF converts the real time form of vehicle dynamic trace into a digital format (further visualized way). Thus, it allows interested parties to extract and read relevant information from a mass number of vehicle dynamics. The information is collected and transferred to a digital format, then BCT enables the verification of this information and captures it into a shared ledger in real time manner. That is, the key features of BCauotSCF enabled by BCT could ensure the trust among interested parties and the availability for data consumers, and form authoritative records, which is the input of a smart contract.

### 4.3. Financing Modes

**Figure 3:**
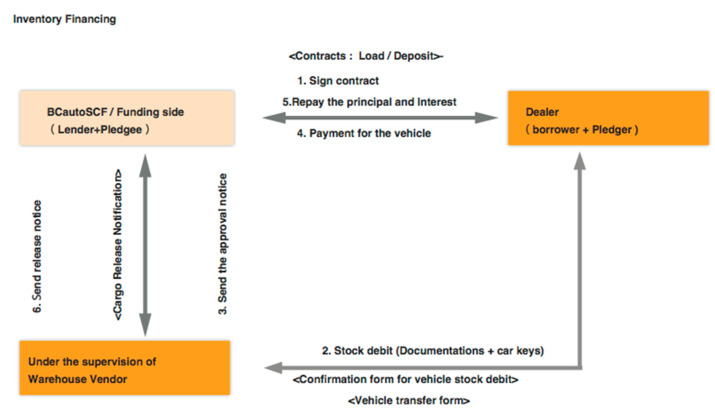
Simplified workflow of inventory financing of BCautoSCF.

**Figure 4:**
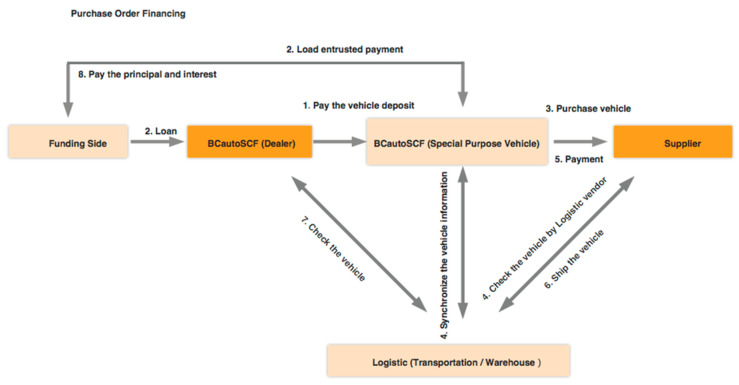
Simplified workflow of purchasing order financing of BCautoSCF.

BCautoSCF provides two SCF modes: “inventory financing” is usually confined to finished goods. In this paper (as shown in Figure 3), the financing party provides funds towards the inventory (i.e., collateral). The financing need depends on the timing of the manufacturing and delivery cycle along its supply chain. It usually targets at secondary dealers, and parallel importer agencies. Compared with auto electronic commercial platforms, our platform is also a good place for inventory financing application to obtain funding to speed up their business expansion. In purchasing order financing (as illustrated in Figure 4), the financing usually completes before the shipment of vehicles. The funds are expected to cover the working capital needs, so that the order can be normally executed. In addition, the supplier, the one that receives the purchase order will be paid the relevant expenses (e.g., raw materials, worker wages). It is on the basis that the order can be used as collateral, and it represents the largest shares in SCF market. 

As illustrated in Figure 3 and Figure 4, it is the fundamental of a SCF transaction that each issued document is unique and it is cryptographically signed by relevant interested parties and associated to a specific transaction. Actually, thanks to the cryptographical mechanism, with the key pairs related to a document and issuer, the financing party could ensure of the authenticity of the document immediately, so that there is no need to perform a manual inspection (time-consuming and unreliable) on the validity of a document. One of the major challenges in the application of SCF programs is the lack of approaches to verify the validity of invoices. By assuring legal validity by reliable mechanism, the overall risk of the financing trade is reduced, which helps all participants in a SCF transaction.

### 4.4. Automated Transaction

**Figure 5:**
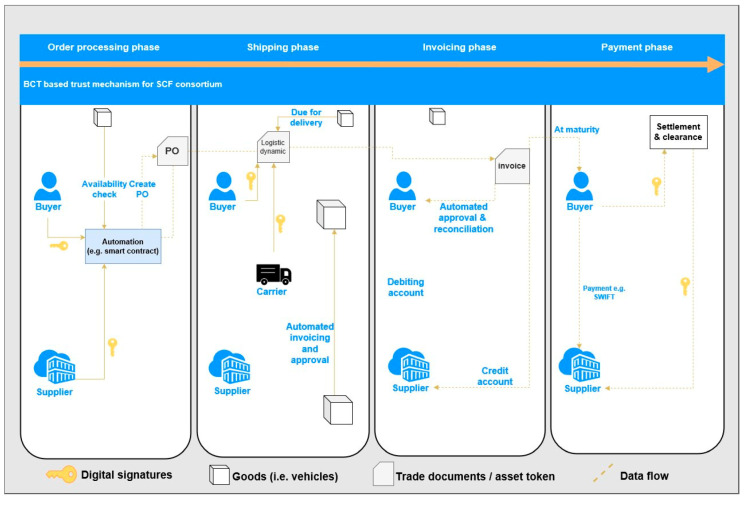
Integrated workflow of SCF in BCautoSCF.

Researchers previously illustrated the methods and principles of using computational terms to describe the legal rules and the consequent structure of financial contracts [22]. Under this framework, Blockchain-driven smart contracts can ensure that logic is executed accurately between untrusted entities without modification by any parties. In this case, smart contracts are designed to automatically execute the state transition of the collaterals/documents/funding ownership among participants in SCF that encode explicit transition rules for shifting the ownership from one to another. Those states are triggered by the realization of certain predefined conditions, such as an event by other parties that may be within or out of their control (for instance “e-contract passed”, “invoice issued”, “vehicle delivered” or “instruction received”). Further, a smart contract can independently schedule various kinds of resources. Instantly, a typical smart contract is designated to raise funds by offering financial services or issuing digital equity. In a Blockchain network, a smart contract is self-executed throughout the whole peer network. According to literatures [22,25], a smart contract could work towards events outside a Blockchain and be applied to physical assets. In order to clearly explain how the key service (i.e., financing) operates, we hide some technical details and simplify the BCautoSCF financing steps to conclude the workflow as illustrated in Figure 5. Document flow could be processed by BCT, which will ensure the authenticity of documents. These vehicles are also uniquely identified, and submitted for the efficient custody, for instance automation program (i.e., smart contracts), that further guarantees that a payment will be handled when certain criterion is reached. In this way, the automatic custody could cause a positive result, such as mutual trust in a commercial relationship, more funds received, and lower transaction costs.

### 4.5. Functional Snapshots

**Figure 6:**
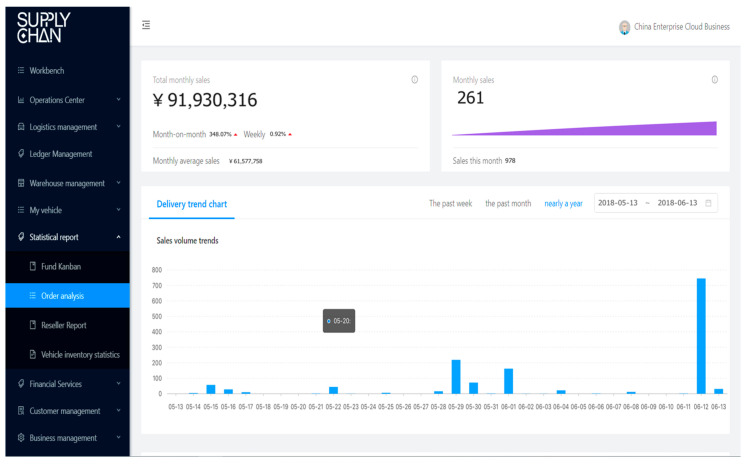
Business dashboard of BCautoSCF platform.

**Figure 7:**
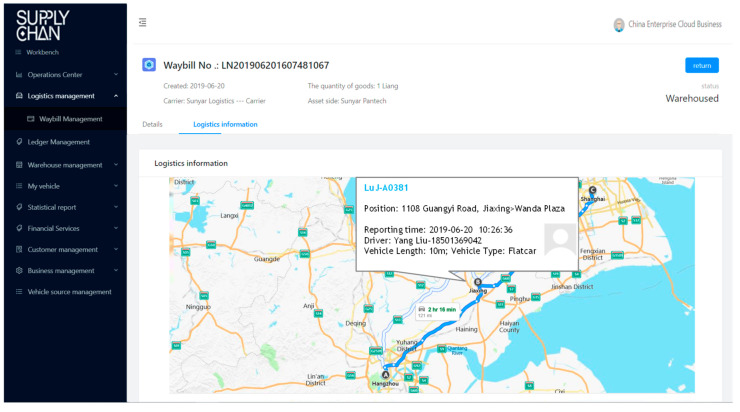
Logistic transportation management module.

**Figure 8:**
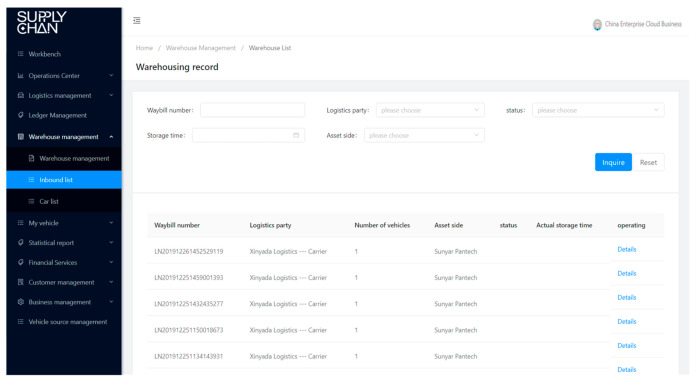
Warehouse management module.

**Figure 9:**
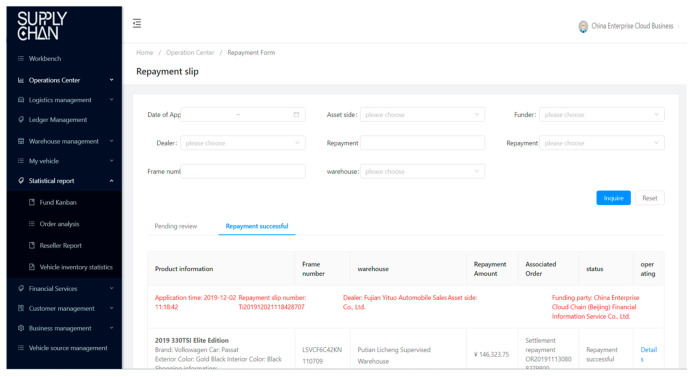
Repayment management module.

BCautoSCF offers equal visibility on transactions and collateral custody information to interested parties in the supply chain, and this offering is reliable, authentic, immutable, and verified only by the granted parties from the SCF consortium. By involving all participants of information and data flow into BCautoSCF and by issuing digitized documents, we can ensure all these activities and documents throughout the supply chain to be equally visible and available to every participant. Every interested party will be provided with information about whom, where, and when issued them or move the vehicles (as illustrated in Figure 6, Figure 7, Figure 8 and Figure 9). This decreases the need for face-to-face communication, avoids human operation errors, and ensures significantly faster transactions in SCF. 

### 4.6. Data Schema 

As illustrated in Figure 1, there is a specific layer containing data flows in our platform; more precisely, these data flows merge the purchasing order details from order management system, vehicle logistic information from transportation management system, cargo warehousing information from warehouse management system, funding flow from multiple sources (e.g., funding provider, platform administrations, etc.), and business information from contracts, agreements, invoices, credit notes, payments, and related documents. Table 1 presents data schema in shared ledgers and the credit-related transaction and schema is described in Table 2.

## 5. Discussion

In order to distinguish our platform from traditional ones, and highlight what we contribute to the research and practical use on SCF, we list these identified shortcomings existing in traditional SCF, and discuss the corresponding solutions enabled by BCautoSCF. In order to outline the opportunities, this paper deals first with the use cases that could help overcome barriers that arise when discussing and presenting the SCF models, and it then successively analyses the impact of the adoption of BCT-driven SCF platform.

In traditional inventory financing, the monitoring of the inventory requires specific service providers, which cause more and more complicated communications and information exchanges among these actors related to a trade. Under this multilateral collaborative environment, the most important concern is the trust towards the information exchanged [19]. BCT-driven activity custody offers an opportunity to bridge various actors throughout the supply chain network, so that the integrity of the logistic status and receipt notes can be maintained. BCautoSCF provides an overlying technical layer upon the physical world for a secure (trade related) information exchange among untrusted interested parties.

There are multiple third parties running different policies. Thus, incorrect and forged documents could increase the risk of an incorrect amount of financing or non-existing collateral from the bank. Risks exist if the collaterals have not been financed yet (i.e., double financed) [24]. Signed by relevant parties and registered into BCautoSCF, the information is available to interested parties. Similar to a bitcoin transaction, the possibility of the same “purchase order” being financed twice (or more) could be eliminated by consulting an authoritative shared ledger that records all transactions related to a specific document or a transaction. Therefore, the financing party could confirm the authenticity of the document and the existence of purchasers.

The legal match between documents and physical goods is the prerequisite for avoiding ownership disputes on collateralized goods. In order to address this issue, the inspection company has to accomplish lots of manual tasks (time-consuming and costly), for instance, manually accounting quantity of the goods, and inspecting the quality of the goods. Providing enough transparency for high amounts of goods is a difficult task, but it is always necessary to prevent possible trade fraudulent behaviors [24,27]. Registering the asset on BCautoSCF could ensure the easily verifiable ownership of the asset in the network. By using smart contract, it can program the transactional steps of the loan agreement between the financing party and supplier. It acts as a trigger for the payment when goods deliver, which can occur as a STP. In this way, we can observe that it significantly increases trade speed, and meanwhile reduces trade costs and the probability of human errors.

Table 3 illustrates the comparison between BCautoSCF with typical online Auto retail platforms from a business perspective (e.g., business strategies, income source). Streaming a vast array of data flows into a distributed ledger brings the possibility for smart contracts to read the relevant information and then process the financing and delivery associated to a specific transaction. Here, we note that the inputs of a smart contract are agreed upon by all these peers in the Blockchain network. The automation of processes is a key driver for the development of the SCF, which directly decides the efficiency and processing speed of supply chain trade. However, due to its initial stage with special concerns in the targeted scenarios, there is limited automation in BCautoSCF. For instance, the self-billing procurement model can be automated to a certain degree, which can be integrated to go into enterprise resource planning system (ERP) and simplifies the approval of payments. Furthermore, a potential speed-up mechanism for the SCF process is to introduce a certain extent of dematerialization and acceleration of processes (i.e., digital invoice) that replaces the paper-based medium and provides faster receipt of the document. Thus, the SCF process automation would be a future research direction. All these procedures should be refined towards the smart contract without human intervention. 

## 6. Conclusions

In this paper, we studied the main points in traditional SCF platforms (always based on centralized mode) and introduced a BCT-driven system design to solve these main points in SCF, and took the Auto retail industry as a trial and successfully developed a BCT-driven SCF platform- BCautoSCF. It performs full custody to all participants, activities, records, and processes of SCF in a reliable, transparent, high-efficiency, and low-cost way. With BCT built-in trust mechanism, it is unnecessary for any third party (e.g., bank) to act as an intermediary. Therefore, the transaction on BCautoSCF could be much faster and more economical as the time-cost is the key factor for SC and logistic service industry. In addition, smart contract is employed to partially automate the workflows in SCF, minimizing human errors and disruption during contract execution. BCautoSCF also introduces a vast array of technologies to enable the full custody of collaterals and information revelation to interested parties in the SC network.

We managed to partially automate SCF workflows by smart contract in the current phase. Although in its initial stage, in the future, we will continue to work towards implementing a full automation of SCF workflow by smart contracts, so that the efficiency of the transaction would be further improved. Actually, due to that fully digital and cryptographically signed delivery documents (e.g., vehicle transfer ticket) exist on the shared ledger, the task will not be a hard one.

## Figures and Tables

**Table 1:** Uniformed stream including four types of data flows.

Category	Elements	Description
Funding flow	Source	Where the funding originates from
	Amount	The credit amount approved in this case
	Mode	The financing mode used in this case
	Receiver	The cooperation, which receives this funding
	Collateral	The collateral used in this case.
	Loan Interest	The interest rate used in this case.
Logistic	Carrier ID	The actual carrier undertakes the shipment
	IoT Garments	IoT garments (e.g., GPS, RFID, etc.) in the shipment
	Shipment	Records the routines, tools, traces, fee, etc.
	Vehicle ID	The identifier of the vehicle in an order, which is always associated with IoT garments.
	Shipper	The owner of the cargo in a shipment.
Document flow	Contract	The contracts associated with this information flow
	Initiator	The sender, who initiates the conversation.
	Receiver	The receiver, who will receive the information
	Type	The type of the information flow
	Participants	The interested parties get involved in this information flow
	Descriptions	The full content of this information flow
Business flow	Status	The current status of this business case
	Collateral	The collateral used in this case
	Dealer	The dealer, which submits this order
	Order	The specification of the order
	Financing	The financing information used in this order

**Table 2:** The definitions and descriptions of credit-related transaction.

Action	Parameters	Parameter Definitions
Registration	Identity	User information including user id, username, password, icon, and other profiles.
	Asset Address List	A list of user’s digital asset in blockchain
	Asset Type	Identifying the type of user’s digital asset
	User Token	User’s token used in transaction
	User Address	User’s digital address in blockchain
Credit Facility	Identity	User information including user id, username, password, icon, etc.
	Receipt Address	The address of credit receipt in blockchain
	Credit Amount	The credit amount approved to the receipt
	CF Record	The record of a credit-facility transaction.
Credit Inquiry	Identity	The identity information of credit receipt
	CI Record	The transaction details of credit inquiry in a record
	User Address	The address of the credit receipt in blockchain
	CF record	The record of a credit-facility transaction.

**Table 3:** Comparative analysis of online auto dealer platforms.

Platform(s)	Type	Business Models and Policies	Income
BCautoSCF	Financing Platform (2B)	It aims to provide cross-industries SCF services to secondary dealers, banks, logistic, and auto sources. It also performs the fourth-party supervision on the auto dealing ecology. It is a professional SCF, and provides in-built auto supply chain service.	Service fee from users
www.chexiang.com	Primary Dealer (2C)	It aims to provide service to direct consumers, and establishes an online daily life service platform, and produces profit from the auto aftermarket. It is a typical O2O electronic commercial platform. It is an equipped supply chain management, but no SCF service is available.	Profit from Auto aftermarket
www.yiautos.com www.chezhency.com	Secondary Dealer (2C)	It focuses on the auto markets of small cities in China, by using the supply chain and advertising, builds several auto chain supermarkets. It purchases best-seller models from auto OEMs, and also receives stock cars from 4S stores. It is positioned between primary dealer and secondary dealer	Price disparities Loan interest Profit of Insurance Finance Profit from Auto aftermarket
www.niuniuqiche.com www.chehang168.com	SCF (2B)	It aims to provide SCF service to secondary dealers, which are always dispersed and lack funding. Only provides SCF service, not includes SC	Profit of SCF Price disparities of funding
www.tangeche.com www.maodou.com	Consumer Finance (2C)	It lowers the threshold for potential consumer by providing consumer-financing service. It runs a sold on a rent basis, which attracts online potential consumers to offline delivery. Finance lease product is not so welcome in small cities in China. It usually gets from finance lease cooperation and dealers, and also receives some unsalable models from auto OMEs. No SCF service, neither supply chain management.	Price disparities Profit of consumer finance Sold on a rent basis

## References

[1] Jüttner U., Maklan S. (2013). Supply chain resilience in the global financial crisis: An empirical study. Supply Chain Manag..

[2] Babich V., Hilary G. (2019). Distributed Ledgers and Operations: What Operations Management Researchers Should Know about Blockchain Technology. Manuf. Serv. Oper. Manag..

[3] Mainelli M.M., Alistair K.L. The Impact and Potential of Blockchain on the Securities Transaction Lifecycle. https://ssrn.com/abstract=2777404.

[4] Kouvelis P., Zhao W. (2012). Supply Chain Finance. The Handbook of Integrated Risk Management in Global Supply Chains.

[5] Gomm M.L. (2010). Supply chain finance: Applying finance theory to supply chain management to enhance finance in supply chains. Int. J. Logist. Res. Appl..

[6] Yli-Huumo J., Ko D., Choi S., Park S., Smolander K. (2016). Where is current research on blockchain technology?—A systematic review. PLoS ONE.

[7] Nakamoto S. (2009). Bitcoin: A Peer-to-Peer Electronic Cash System. https://bitcoin.org/bitcoin.pdf.

[8] Schollmeier R. A Definition of Peer-to-Peer Networking for the Classification of Peer-to-Peer Architectures and Applications. Proceedings of the First International Conference on Peer-to-Peer Computing.

[9] Montecchi M., Plangger K., Etter M. (2019). It’s real, trust me! Establishing supply chain provenance using blockchain. Bus. Horiz..

[10] Christidis K., Devetsikiotis M. (2016). Blockchains and smart contracts for the Internet of things. IEEE Access.

[11] Hofmann E., Strewe U.M., Bosia N. (2017). Supply chain finance and blockchain technology. O’Leary, D.E. ConFigureuring blockchain architectures for transaction information in blockchain consortiums: The case of accounting and supply chain systems. Intell. Syst. Account. Financ. Manag..

[12] Wang Y., Singgih M., Wang J., Rit M. (2019). Making sense of blockchain technology: How will it transform supply chains?. Int. J. Prod. Econ..

[13] Choi T.M., Wen X., Sun S.T., Chung S.H. (2019). The mean-variance approach for global supply chain risk analysis with air logistics in the blockchain technology era. Trans. Res. Part E.

[14] Pournader M., Shi Y., Seuring S., Koh S.C. (2019). Blockchain applications in supply chains, transport and logistics: A systematic review of the literature. Int. J. Prod. Res..

[15] Flood N., Goodenough O. Contracts as Automaton: The Computational Representation of Financial Agreement. https://financialresearch.gov/working-papers/files/OFRwp-2015-04_Contract-as-Automaton-The-Computational-Representation-of-Financial-Agreements.pdf.

[16] Yang F., Zhou W., Wu Q., Long R., Xiong N.N., Zhou M. (2019). Delegated Proof of Stake with Downgrade: A Secure and Efficient Blockchain Consensus Algorithm with Downgrade Mechanism. IEEE Access.

[17] Chen L., Xu L., Shah N., Gao Z., Lu Y., Shi W. On Security Analysis of Proof-of-Elapsed-Time (PoET). Proceedings of the International Symposium on Stabilization Safety and Security of Distributed Systems.

[18] Szabo N. (1994). Smart Contracts. http://szabo.best.vwh.net/smart.contracts.html.

[19] Buterin V. (2013). A Next Generation Smart Contract and Decentralized Application Platform. https://ethereumbuilders.gitbooks.io/guide/content/en/whitepaper.html.

[20] Babich V., Hilary G. (2019). Blockchain and other Distributed Ledger Technologies in Operations. Found. Trends® Technol. Inf. Oper. Manag..

[21] Choi T., He Y. (2019). Peer-to-peer collaborative consumption for fashion products in the sharing economy: Platform operations. Trans. Res. Part E.

[22] Yang C. (2019). Maritime shipping digitalization: Blockchain-based technology applications, future improvements, and intention to use. Trans. Res. Part E.

[23] Fan X. Scalable Practical Byzantine Fault Tolerance with Short-Lived Signature Schemes. Proceedings of the 28th Annual International Conference on Computer Science and Software Engineering.

[24] Marak Z., Pillai D. (2019). Factors, Outcome, and the Solutions of Supply Chain Finance: Review and the Future Directions. J. Risk Financ. Manag..

[25] Dolgui A., Ivanov D., Potryasaev S.A., Sokolov B., Ivanova M., Werner F. (2019). Blockchain-oriented dynamic modelling of smart contract design and execution in the supply chain. Int. J. Prod. Res..

[26] Choi T., Luo S. (2019). Data quality challenges for sustainable fashion supply chain operations in emerging markets: Roles of blockchain, government sponsors and environment taxes. Trans. Res. Part E.

[27] Wang F., Yang X., Zhuo X., Xiong M. (2019). Joint logistics and financial services by a 3PL firm: Effects of risk preference and demand volatility. Trans. Res. Part E.

[28] Choi T., Feng L., Li R. (2019). Information disclosure structure in supply chains with rental service platforms in the blockchain technology era. Int. J. Prod. Econ..

